# Supplementary material for: The interaction of ammonia and manganese in abnormal metabolism of minimal hepatic encephalopathy: A comparison metabolomics study
Source: PLoS One. 2023 Aug 4;18(8):e0289688. doi: 10.1371/journal.pone.0289688 (PMC10403054; doi:10.1371/journal.pone.0289688)
Supplement: S1 Table — (DOCX) [file pone.0289688.s001.docx]

**Supplementary Table 1. Metabolic pathways of the the key metabolites involved in the CHA and MHE rats**

| KEGG ID | Entry type | KEGG name | P |
| --- | --- | --- | --- |
| rno00220 | pathway | Arginine biosynthesis - Rattus norvegicus (ra... | <0.001 |
| rno00250 | pathway | Alanine, aspartate and glutamate metabolism -... | <0.001 |
| rno00290 | pathway | Valine, leucine and isoleucine biosynthesis -... | 0.027 |
| rno00330 | pathway | Arginine and proline metabolism - Rattus norv... | 0.026 |
| rno00430 | pathway | Taurine and hypotaurine metabolism - Rattus n... | <0.001 |
| rno00620 | pathway | Pyruvate metabolism - Rattus norvegicus (rat) | <0.001 |
| rno04730 | pathway | Long-term depression - Rattus norvegicus (rat... | 0.034 |
| rno05144 | pathway | Malaria - Rattus norvegicus (rat) | 0.038 |
| rno05330 | pathway | Allograft rejection - Rattus norvegicus (rat) | 0.038 |
| rno05332 | pathway | Graft-versus-host disease - Rattus norvegicus... | 0.038 |
| M00027 | module | GABA (gamma-Aminobutyrate) shunt | <0.001 |
| M00029 | module | Urea cycle | <0.001 |
| M00106 | module | Conjugated bile acid biosynthesis, cholate =>... | 0.021 |
| M00119 | module | Pantothenate biosynthesis, valine/L-aspartate... | 0.014 |
| M00131 | module | Inositol phosphate metabolism, Ins(1,3,4,5)P4... | 0.024 |
| M00134 | module | Polyamine biosynthesis, arginine => ornithine... | <0.001 |
| M00169 | module | CAM (Crassulacean acid metabolism), light | 0.025 |
| M00171 | module | C4-dicarboxylic acid cycle, NAD - malic enzym... | 0.011 |
| M00844 | module | Arginine biosynthesis, ornithine => arginine | <0.001 |
| M00845 | module | Arginine biosynthesis, glutamate => acetylcit... | <0.001 |
| C00022 | compound | Pyruvate | <0.001 |
| C00025 | compound | L-Glutamate | <0.001 |
| C00026 | compound | 2-Oxoglutarate | 0.006 |
| C00041 | compound | L-Alanine | <0.001 |
| C00062 | compound | L-Arginine | <0.001 |
| C00064 | compound | L-Glutamine | <0.001 |
| C00077 | compound | L-Ornithine | 0.008 |
| C00123 | compound | L-Leucine | <0.001 |
| C00137 | compound | myo-Inositol | <0.001 |
| C00245 | compound | Taurine | <0.001 |
| C00256 | compound | (R)-Lactate | <0.001 |
| C00327 | compound | L-Citrulline | <0.001 |
| C00407 | compound | L-Isoleucine | <0.001 |
| C00533 | compound | Nitric oxide | 0.038 |
| C00671 | compound | (S)-3-Methyl-2-oxopentanoic acid | 0.026 |
| C01235 | compound | alpha-D-Galactosyl-(1->3)-1D-myo-inositol | 0.034 |
| C05933 | compound | N(omega)-Hydroxyarginine | 0.049 |
| C20966 | compound | 3-(2E)-4-Amino-4-oxobut-2-enoylamino-L-al... | 0.043 |
