# Supplementary material for: The interaction of ammonia and manganese in abnormal metabolism of minimal hepatic encephalopathy: A comparison metabolomics study
Source: PLoS One. 2023 Aug 4;18(8):e0289688. doi: 10.1371/journal.pone.0289688 (PMC10403054; doi:10.1371/journal.pone.0289688)
Supplement: S2 Table — (DOCX) [file pone.0289688.s002.docx]

**Supplementary Table 2. Metabolic pathways of the the key metabolites involved in the CHM and MHE rats**

| KEGG ID | Entry type | KEGG name | P |
| --- | --- | --- | --- |
| rno00052 | pathway | Galactose metabolism - Rattus norvegicus (rat... | 0.045 |
| rno00410 | pathway | beta-Alanine metabolism - Rattus norvegicus (... | 0.034 |
| rno00430 | pathway | Taurine and hypotaurine metabolism - Rattus n... | <0.001 |
| rno00562 | pathway | Inositol phosphate metabolism - Rattus norveg... | 0.017 |
| rno00620 | pathway | Pyruvate metabolism - Rattus norvegicus (rat) | <0.001 |
| rno04727 | pathway | GABAergic synapse - Rattus norvegicus (rat) | 0.030 |
| M00027 | module | GABA (gamma-Aminobutyrate) shunt | <0.001 |
| M00106 | module | Conjugated bile acid biosynthesis, cholate =>... | 0.001 |
| M00131 | module | Inositol phosphate metabolism, Ins(1,3,4,5)P4... | 0.002 |
| M00135 | module | GABA biosynthesis, eukaryotes, putrescine => ... | 0.002 |
| M00168 | module | CAM (Crassulacean acid metabolism), dark | 0.019 |
| M00169 | module | CAM (Crassulacean acid metabolism), light | 0.006 |
| M00172 | module | C4-dicarboxylic acid cycle, NADP - malic enzy... | 0.021 |
| C00022 | compound | Pyruvate | 0.006 |
| C00123 | compound | L-Leucine | <0.001 |
| C00137 | compound | myo-Inositol | <0.001 |
| C00233 | compound | 4-Methyl-2-oxopentanoate | 0.015 |
| C00245 | compound | Taurine | <0.001 |
| C00256 | compound | (R)-Lactate | <0.001 |
| C00334 | compound | 4-Aminobutanoate | <0.001 |
| C00492 | compound | Raffinose | 0.039 |
| C00593 | compound | Sulfoacetaldehyde | 0.034 |
| C01235 | compound | alpha-D-Galactosyl-(1->3)-1D-myo-inositol | 0.004 |
