# Supplementary material for: The interaction of ammonia and manganese in abnormal metabolism of minimal hepatic encephalopathy: A comparison metabolomics study
Source: PLoS One. 2023 Aug 4;18(8):e0289688. doi: 10.1371/journal.pone.0289688 (PMC10403054; doi:10.1371/journal.pone.0289688)
Supplement: S3 Table — (DOCX) [file pone.0289688.s003.docx]

**Supplementary Table 3. Metabolic pathways of the the key metabolites involved in the MHE rats**

| KEGG ID | Entry type | KEGG name | P |
| --- | --- | --- | --- |
| rno00220 | pathway | Arginine biosynthesis - Rattus norvegicus (ra... | <0.001 |
| rno00250 | pathway | Alanine, aspartate and glutamate metabolism -... | <0.001 |
| rno00260 | pathway | Glycine, serine and threonine metabolism - Ra... | 0.023 |
| rno00290 | pathway | Valine, leucine and isoleucine biosynthesis -... | 0.043 |
| rno00330 | pathway | Arginine and proline metabolism - Rattus norv... | 0.008 |
| rno00430 | pathway | Taurine and hypotaurine metabolism - Rattus n... | <0.001 |
| rno00620 | pathway | Pyruvate metabolism - Rattus norvegicus (rat) | <0.001 |
| rno00970 | pathway | Aminoacyl-tRNA biosynthesis - Rattus norvegic... | 0.038 |
| rno04727 | pathway | GABAergic synapse - Rattus norvegicus (rat) | 0.042 |
| M00027 | module | GABA (gamma-Aminobutyrate) shunt | <0.001 |
| M00029 | module | Urea cycle | <0.001 |
| M00047 | module | Creatine pathway | 0.033 |
| M00090 | module | Phosphatidylcholine (PC) biosynthesis, cholin... | 0.007 |
| M00092 | module | Phosphatidylethanolamine (PE) biosynthesis, e... | 0.037 |
| M00119 | module | Pantothenate biosynthesis, valine/L-aspartate... | 0.026 |
| M00131 | module | Inositol phosphate metabolism, Ins(1,3,4,5)P4... | 0.043 |
| M00134 | module | Polyamine biosynthesis, arginine => ornithine... | <0.001 |
| M00135 | module | GABA biosynthesis, eukaryotes, putrescine => ... | 0.031 |
| M00171 | module | C4-dicarboxylic acid cycle, NAD - malic enzym... | 0.026 |
| M00844 | module | Arginine biosynthesis, ornithine => arginine | <0.001 |
| M00845 | module | Arginine biosynthesis, glutamate => acetylcit... | 0.001 |
| C00002 | compound | ATP | 0.030 |
| C00008 | compound | ADP | 0.041 |
| C00009 | compound | Orthophosphate | 0.038 |
| C00022 | compound | Pyruvate | 0.001 |
| C00025 | compound | L-Glutamate | <0.001 |
| C00026 | compound | 2-Oxoglutarate | 0.014 |
| C00041 | compound | L-Alanine | <0.001 |
| C00062 | compound | L-Arginine | <0.001 |
| C00064 | compound | L-Glutamine | <0.001 |
| C00077 | compound | L-Ornithine | 0.005 |
| C00123 | compound | L-Leucine | <0.001 |
| C00137 | compound | myo-Inositol | <0.001 |
| C00188 | compound | L-Threonine | <0.001 |
| C00245 | compound | Taurine | <0.001 |
| C00256 | compound | (R)-Lactate | <0.001 |
| C00327 | compound | L-Citrulline | <0.001 |
| C00334 | compound | 4-Aminobutanoate | <0.001 |
| C00407 | compound | L-Isoleucine | <0.001 |
| C00588 | compound | Choline phosphate | <0.001 |
