# Supplementary material for: The interaction of ammonia and manganese in abnormal metabolism of minimal hepatic encephalopathy: A comparison metabolomics study
Source: PLoS One. 2023 Aug 4;18(8):e0289688. doi: 10.1371/journal.pone.0289688 (PMC10403054; doi:10.1371/journal.pone.0289688)
Supplement: S4 Table — (DOCX) [file pone.0289688.s004.docx]

**Supplementary Table 4. The chemical shifts and chemical structure for the 45 metabolites characterized by NMR spectroscopy**

| Key | Metabolites | δ 1H (ppm) and multiplicity* | HMDB | PubChem | KEGG | SMILES |
| --- | --- | --- | --- | --- | --- | --- |
| 1 | L-Isoleucine | 0.94(t), 0.99(d), 1.26(m), 1.46(m), 1.97(m), 3.68(d) | HMDB0000172 | 6306 | C00407 | CC[C@H](C)[C@@H](C(=O)O)N |
| 2 | L-Leucine | 0.96(d), 0.97(d), 1.67(m), 1.70(m), 1.73(m), 3.72(dd) | HMDB0000687 | 6106 | C00123 | CC(C)C[C@@H](C(=O)O)N |
| 3 | L-Valine | 0.97(d), 1.01(d), 2.27(m), 3.60(d) | HMDB0000883 | 6287 | C00183 | CC(C)[C@@H](C(=O)O)N |
| 4 | alpha-Hydroxyisobutyric Acid | 1.34(s) | HMDB0000729 | 11671 |  | CC(C)(C(=O)O)O |
| 5 | L-Threonine | 1.20(d), 2.30(dd) , 2.39(dd) , 4.14(m) | HMDB0000167 | 6288 | C00188 | C[C@H]([C@@H](C(=O)O)N)O |
| 6 | L-Lactic acid | 1.33(d), 4.11(q) | HMDB0000190 | 61503 | C00256 | C[C@@H](C(=O)O)O |
| 7 | L-Alanine | 1.49(d), 3.78(q) | HMDB0000161 | 5950 | C00041 | C[C@@H](C(=O)O)N |
| 8 | L-Lysine | 1.44(m), 1.51(m), 1.73(m), 1.89(m), 1.92(m), 3.03(t), 3.77(t), | HMDB0000182 | 5962 | C00047 | C(CCN)C[C@@H](C(=O)O)N |
| 9 | Gamma-Aminobutyric acid | 1.91(m), 2.30(t), 3.01(m) | HMDB0000112 | 223130 | C00334 | C(CC(=O)O)CN |
| 10 | L-Arginine | 1.66(m), 1.73(m), 1.91(m), 1.92(m), 3.25(t), 3.78(t), | HMDB0000517 | 6322 | C00062 | C(C[C@@H](C(=O)O)N)CN=C(N)N |
| 11 | Acetic Acid | 1.92(s) | HMDB0000042 | 176 | C00033 | CC(=O)O |
| 12 | Pyroglutamic Acid | 2.02(s), 2.50(dd), 2.69(dd), 4.39(m) | HMDB0000267 | 7405 | C01879 | C1CC(=O)N[C@@H]1C(=O)O |
| 13 | L-Glutamic Acid | 2.06(m), 2.14(m), 2.34(m), 2.37(m), 3.76(dd) | HMDB0000148 | 33032 | C00025 | C(CC(=O)O)[C@@H](C(=O)O)N |
| 14 | L-Glutamine | 2.14(m), 2.15(m), 2.44(m), 2.47(m), 3.78(t), 6.85(s), | HMDB0000641 | 5961 | C00064 | C(CC(=O)N)[C@@H](C(=O)O)N |
| 15 | Citric acid | 2.51(d), 2.70(d) | HMDB0000094 | 311 | C00158 | C(C(=O)O)C(CC(=O)O)(C(=O)O)O |
| 16 | L-Aspartic acid | 2.70(dd), 2.81(dd), 3.90(dd) | HMDB0000191 | 5960 | C00049 | C([C@@H](C(=O)O)N)C(=O)O |
| 17 | Creatine | 3.04(s), 3.93(s) | HMDB0000064 | 586 | C00300 | CN(CC(=O)O)C(=N)N |
| 18 | Choline | 3.21(s), 3.53(m), 4.07(m) | HMDB0000097 | 305 | C00114 | C[N+](C)(C)CCO |
| 19 | Phosphorylcholine | 3.21(s), 3.60/(m), 4.17(m) | HMDB0001565 | 8691 | C00588 | C[N+](C)(C)CCOP(=O)(O)O |
| 20 | L-Cysteine | 3.02(dd), 3.70(s), 3.95(t) | HMDB0000574 | 5862 | C00097 | C([C@@H](C(=O)O)N)S |
| 21 | myo-Inositol | 3.29(t), 3.54(dd), 3.63(t), 4.07(m) | HMDB0000211 |  | C00137 | O[C@H]1[C@H](O)[C@@H](O)[C@H](O)[C@H](O)[C@@H]1O |
| 22 | Taurine | 3.27(t), 3.42(t) | HMDB0000251 | 1123 | C00245 | C(CS(=O)(=O)O)N |
| 23 | Ethanolamine | 3.13(t), 3.81(t) | HMDB0000149 | 700 | C00189 | C(CO)N |
| 24 | L-Phenylalanine | 3.13(dd), 3.29(dd), 4.00(dd), 7.33(m), 7.38(m), 7.43(m) | HMDB0000159 | 6140 | C00079 | C1=CC=C(C=C1)C[C@@H](C(=O)O)N |
| 25 | Glycine | 3.56(s) | HMDB0000123 | 750 | C00037 | C(C(=O)O)N |
| 26 | Uracil | 5.81(d), 7.55(d) | HMDB0000300 | 1174 | C00106 | C1=CNC(=O)NC1=O |
| 27 | Threonic Acid | 3.71(dd), 4.25(m) | HMDB0000943 | 151152 | C01620 | C([C@H]([C@@H](C(=O)O)O)O)O |
| 28 | Guanidoacetic acid | 3.78(dd) | HMDB0000128 | 763 | C00581 | C(C(=O)O)N=C(N)N |
| 29 | L-Tyrosine | 6.52(s) | HMDB0000158 | 6057 | C00082 | C1=CC(=CC=C1C[C@@H](C(=O)O)N)O |
| 30 | L-Asparagine | 2.84(dd), 3.84(dd) | HMDB0000168 | 6267 | C00152 | C([C@@H](C(=O)O)N)C(=O)N |
| 31 | Carnosine | 3.14(dd), 3.24(dd), 3.98(dd), 7.10(s), 7.90(s) | HMDB0000033 | 439224 | C00386 | C1=C(NC=N1)C[C@@H](C(=O)O)NC(=O)CCN |
| 32 | Nicotinuric Acid | 3.0(d), 3.24(t), 7.05(s), 8.01(s) | HMDB0003269 | 68499 | C05380 | C1=CC(=CN=C1)C(=O)NCC(=O)O |
| 33 | Guanosine monophosphate | 8.31(s) | HMDB0001397 | 6804 | C00144 | C1=NC2=C(N1[C@H]3[C@@H]([C@@H]([C@H](O3)COP(=O)(O)O)O)O)NC(=NC2=O)N |
| 34 | Hypoxanthine | 8.19(s), 8.21(s) | HMDB0000157 | 790 | C00262 | C1=NC2=C(N1)C(=O)N=CN2 |
| 35 | L-Serine | 3.84(t), 3.94(dd) | HMDB0000187 | 5951 | C00065 | C([C@@H](C(=O)O)N)O |
| 36 | Glycerol | 3.43(dd), 3.60(d), 3.85(m) | HMDB0000131 | 753 | C00116 | C(C(CO)O)O |
| 37 | L-Proline | 2.02(m), 3.32(dt), 4.12(dd) | HMDB0000162 | 145742 | C00148 | C1C[C@H](NC1)C(=O)O |
| 38 | L-Methionine | 2.12(s), 2.63(t), 3.85(t) | HMDB0000696 | 6137 | C00073 | CSCC[C@@H](C(=O)O)N |
| 39 | Citrulline | 1.53(tt), 3.13(d), 3.85(t) | HMDB0000904 | 9750 | C00327 | C(C[C@@H](C(=O)O)N)CNC(=O)N |
| 40 | Malic Acid | 2.64(dd), 2.75(dd) | HMDB0000744 | 525 | C03668 | C(C(C(=O)O)O)C(=O)O |
| 41 | Beta-D-Glucose 6-phosphate | 3.49(t), 3.71(d), 4.02(dd) | HMDB0003498 | 439427 | C01172 | C([C@@H]1[C@H]([C@@H]([C@H]([C@@H](O1)O)O)O)O)OP(=O)(O)O |
| 42 | Acetylglycine | 2.04(s), 3.75(s) | HMDB0000532 | 10972 |  | CC(=O)NCC(=O)O |
| 43 | Argininosuccinic Acid | 2.79(dd), 3.76(dd) | HMDB0000052 | 16950 | C03406 | C(C[C@@H](C(=O)O)N)CN=C(N)N[C@@H](CC(=O)O)C(=O)O |
| 44 | Methylguanidine | 2.83(s), 3.37(s) | HMDB0001522 | 10111 | C02294 | CN=C(N)N |
| 45 | Ornithine | 1.94(d), 3.04(t) | HMDB0000214 | 6262 | C00077 | C(C[C@@H](C(=O)O)N)CN |
