# Supplementary figures and images for: The interaction of ammonia and manganese in abnormal metabolism of minimal hepatic encephalopathy: A comparison metabolomics study
Source: PLoS One. 2023 Aug 4;18(8):e0289688. doi: 10.1371/journal.pone.0289688 (PMC10403054; doi:10.1371/journal.pone.0289688)

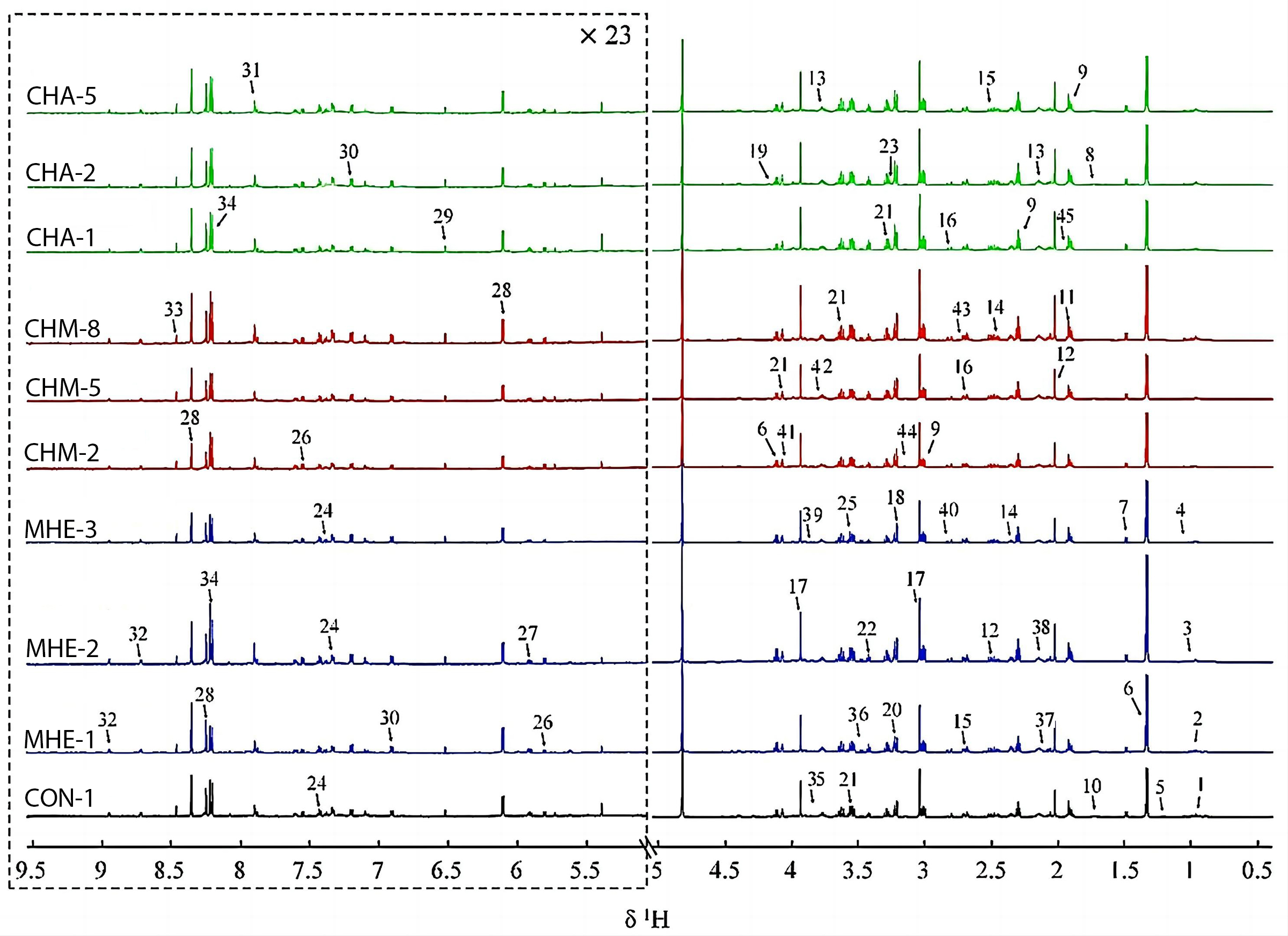

Supplement: S1 Fig — 1, L-Isoleucine; 2, L-Leucine; 3, L-Valine; 4, alpha-Hydroxyisobutyric Acid; 5, L-Threonine; 6, L-Lactic acid; 7, L-Alanine; 8, L-Lysine; 9, Gamma-Aminobutyric acid; 10, L-Arginine; 11, Acetic Acid; 12, Pyroglutamic Acid; 13, L-Glutamic Acid; 14, L-Glutamine; 15, Citric acid; 16, L-Aspartic acid; 17, Creatine; 18, Choline; 19, Phosphorylcholine; 20, L-Cysteine; 21, myo-Inositol; 22, Taurine; 23, Ethanolamine; 24, L-Phenylalanine; 25, Glycine; 26, Uracil; 27, Threonic Acid; 28, Guanidoacetic acid; 29, L-Tyrosine; 30, L-Asparagine; 31, Carnosine; 32, Nicotinuric Acid; 33, Guanosine monophosphate; 34, Hypoxanthine; 35, L-Serine; 36, Glycerol; 37, L-Proline; 38, L-Methionine; 39, Citrulline; 40, Malic Acid; 41, Beta-D-Glucose 6-phosphate; 42, Acetylglycine; 43, Argininosuccinic Acid; 44, Methylguanidine; 45, Ornithine. (TIF) [file pone.0289688.s005.tif]
